# A Case Series and Literature Review on Zanubrutinib Therapy for the Treatment of Relapsed/Refractory Immune Thrombocytopenia

**DOI:** 10.1002/cnr2.70152

**Published:** 2025-02-19

**Authors:** Bingjie Ding, Mengjuan Li, Liu Liu, Xuewen Song, Yuanyuan Zhang, Ao Xia, Jingyuan Liu, Hu Zhou

**Affiliations:** ^1^ Department of Hematology The Affiliated Cancer Hospital of Zhengzhou University & Henan Cancer Hospital, Hemostasis and Thrombosis Diagnostic Engineering Research Center of Henan Province Zhengzhou China; ^2^ Department of Hematology First Affiliated Hospital of Zhengzhou University Zhengzhou China

**Keywords:** BTK inhibitor, immune thrombocytopenia, refractory, relapse

## Abstract

**Background:**

Immune thrombocytopenia (ITP) is an acquired autoimmune disease characterised by low platelet count. Treatment discontinuation or heterogeneity in the pathogenesis of ITP heightens the occurrence of relapsed or refractory ITP. Bruton's tyrosine kinase (BTK) has emerged as a promising target for autoimmune disorders.

**Case:**

In this case series, we have explored the efficacy and safety of Bruton's tyrosine kinase inhibitors (BTKi) in treating relapsed/refractory ITP, by retrospective analysis of the diagnostic history and efficacy of four patients with relapsed/refractory ITP who attended the Affiliated Cancer Hospital of Zhengzhou University and were treated with BTKi. All four patients received > 4 lines of ITP treatment and did not respond to splenectomy or other interventions before and after treatment with BTK inhibitor. After adjusting to the treatment with BTKi, one patient achieved a complete response, and two patients achieved a partial response. All three patients achieved sustained remission with platelet counts of > 50 × 10^9^/L maintained for 1045, 390 and 334 days, respectively. Another patient died of intracranial haemorrhage due to a decline in the platelet count after discontinuation of the drug, and the duration of sustained remission before discontinuation of the drug was 120 days. Four patients had no significant abnormalities in the functions of the liver and kidney monitored during the treatment period.

**Conclusion:**

For patients with relapsed/refractory ITP, BTK inhibitor therapy can be considered as an option, with promising preliminary efficacy and safety. However, more clinical trials are needed to verify the exact data.

## 1 Introduction

Immune thrombocytopenia (ITP) is an acquired autoimmune disease characterised by low platelet count (< 100 × 10^9^/L), which occurs due to impaired production or immune‐mediated destruction of platelets [1]. ITP causes bleeding in the skin and the mucous membranes and even leads to fatal intracranial hemorrhage. The annual incidence of ITP is about ~5/100 000 in children and ~2/100 000 in adults and is more prevalent in geriatric females [2]. Although benign in nature, the Global Burden of Disease Survey on ITP (ITP World Impact Survey, I‐WISh) considered ITP as a disorder that could severely impact health‐related quality of life (HRQoL) [3]. The therapeutic options for ITP include intravenous immune globulin (IVIG), glucocorticoids (GC), including methylprednisolone, prednisolone (Pred), dexamethasone (DXM), mycophenolate mofetil and anti‐RhD immunoglobulin (anti‐D) [4], which focuses mainly on reducing bleeding by interfering with platelet destruction to rapidly increase platelet counts [1, 5, 6, 7]. Treatment discontinuation or heterogeneity in the pathogenesis of ITP heightens the occurrence of relapsed or refractory ITP.

At present, second‐ and third‐line therapies are widely employed in the treatment of relapsed or refractory ITP. Second‐line therapy includes thrombopoietic agents (such as Recombinant human TPO [rhTPO], TPO receptor agonists [TPO‐RA], rituximab [RTX] and rhTPO in combination with rituximab), immunosuppressants (such as azathioprine) [8] and splenectomy [9]. Third‐line therapy includes low‐dose decitabine (DAC), all‐trans retinoic acid (ATRA) in combination with danazol and immunosuppressive therapies (e.g., vincristine and cyclosporine A). Apart from autoantibody‐mediated platelet destruction, IgG antibodies also target platelets via Fcγ receptors (present on reticular cells and Kupffer cells) and Ashwell‐Morell receptors (present on hepatocytes). In this context, novel therapies are being explored to improve the rate of sustained remission [6, 10, 11].

Bruton's tyrosine kinase (BTK) is widely expressed in many cells and plays a key role in B cell maturation, antibody production and Fcy receptor‐mediated signalling pathways [12, 13]. Inhibition of BTK, especially in autoimmune disorders, has the potential to decrease Fcy receptor‐mediated macrophage function and reduce autoantibody production. The activity of selective BTK inhibitors in collagen‐induced arthritis and other rodent models of inflammation has been demonstrated in several studies [14, 15, 16].A study by Xu et al. explored the effects of BTKi in rodent models of arthritis and immune hypersensitivity demonstrating significant anti‐inflammatory and bone‐protective effects [17]. Another recent study by Akasaka et al. demonstrated the therapeutic effects of a different BTKi in experimental models for arthritis and demonstrated effective inhibition of BTK enzyme activity and suppression of various immune‐related signaling pathways [18]. These evidences provide a rationale for the use of BTKi in autoimmune diseases.

Zanubrutinib, a second‐generation irreversible BTKi, inhibits the kinase activity by binding covalently to the cysteine residue in the BTK active binding site [19]. It has more target occupancy and less off‐target binding compared to the first clinically effective covalent BTK inhibitor, Ibrutinib [19, 20]. The enhanced selectivity of Zanubrutinib may be due to its higher reversible binding capability. It showed higher values of IC50 and no inhibition of kinase activities compared to Ibrutinib. The oral absorption and target occupancy of Zanubrutinib is also greater than Ibrutinib. Zanubrutinib has complete BTK occupancy in mononuclear cells. Cardiac side effects and atrial fibrillation were reported to be lower with Zanubrutinib treatment as compared to Ibrutinib [21]. Incidence of other adverse events, such as bleeding and haemorrhage, is lower with Zanubrutinib [22].

Herein, we report four cases treated with BTK inhibitor (BTKi), Zanubrutinib for the treatment of relapsed/refractory ITP. We also conducted a review of the relevant literature to further explore the clinical feasibility of BTKi for treating ITP.

## 2 Case Reports

### 2.1 Case 1

Patient 1, a 61‐year‐old male, diagnosed with thrombocytopenia for > 4 years without any family history of the condition, was hospitalised in August 2022. The patient presented with gingival bleeding with petechiae on both lower limbs in May 2018. In July 2018, the patient's routine blood test revealed white blood cells (WBC) of 4.6 × 10^9^/L, haemoglobin of 119 g/L and platelet count of 3 × 10^9^/L. The bone marrow smear showed active bone marrow hyperplasia, 127 megakaryocytes, 25 classified megakaryocytes, 22 granulocytes, 2 plate giants and 1 naked nucleus. Bone marrow biopsy showed bone marrow hyperplasia as normal (about 45%), with many megakaryocytes and predominantly lobulated nuclei. Reticular fiber staining was seen as MF‐0 grade. Upon further examination, the patient was diagnosed with ITP and was treated with a high dose of DXM ([HD‐DXM] 40 mg, 4 days). The patient was discharged from the hospital after a repeat platelet test (66 × 10^9^/L).

After discharge, oral pred was gradually tapered off and gingival bleeding reappeared on May 1, 2021, and a gradual decrease in platelet count (3 × 10^9^/L) and was transfused with platelets. Upon hospital discharge, oral methylprednisolone (20 mg, q12h) was prescribed 1 month after the discontinuation of oral pred. Between May 2021 and August 2022, the patient underwent platelet transfusions and received various treatments. The results are detailed in Table 1.

**TABLE 1:** Patient characteristics.

Case number	Timeline	Clinical events	Laboratory data	Treatment	Outcome
Case 1	25 May 2021	Patient re‐hospitalised	Platelet count: 1 × 10^9^/L	Platelet transfusion HD‐DXM, IVIG, and Eltrombopag	No improvement in condition
	3 June 2021	Admitted to hospital with thrombocytopenia	Platelet count: 2 × 10^9^/L	Rituximab (0.1 g, q.wk × 4 times) Oral Chinese medicine	Rituximab improved the platelet count to 60 × 10^9^/L Oral Chinese medicine resulted in unsatisfactory results
	July 2022	Thrombocytopenia re‐diagnosed	Platelet count: 1 × 10^9^/L	DAC 6 mg × 3 days Herombopag 5 mg q.d	No significant increase in platelet count
	August 2022	Admitted to hospital with thrombocytopenia	Platelet count: 6 × 10^9^/L	DAC (6 mg × 3 days + pred) and Herombopag along with HD‐DXM, IVIG, and Avatrombopag	No significant improvement in the platelet count
Case 2	2 May 2018 and 23 May 2018	Lymphoma	Platelet count: 25 × 10^9^/L	R‐CHOP + Ibrutinib (280 mg, q.d)	Platelet count repeatedly decreased
	4 July 2018	Lymphoma	Platelet count: 30 × 10^9^/L	Peripheral blood haematopoietic stem cells infusion	On 2 September 2018, the platelet count was 6 × 10^9^/L and Neutrophil count: 0.59 × 10^9^/L The results of bone marrow smear, biopsy and gene examination were completed, and ITP was still diagnosed
	18 December 2018	Thrombocytopenia	Platelet count: 8 × 10^9^/L	Amifostine (0.4 g d1‐d5 discontinued for 2 days) was given for 4 cycles	No significant improvement in the platelet count
	18 September 2019	Thrombocytopenia	Platelet count: 5 × 10^9^/L	RTX (100 mg, q.wk × 4 times)	No sign of lymphoma recurrence
	June 2021	Intracranial haemorrhage due to thrombocytopenia	Platelet count: 1 × 10^9^/L	HD‐DXM, pred, IVIG, RTX, and Eltrombopag	Limited effectiveness
Case 3	July 2017	Low platelet count	Platelet count: 30 × 10^9^/L	Pred (40 mg) for > 2 months and was gradually tapered off	The platelet count rose to normal levels and then decreased gradually
	October 2017	Low platelet count	Platelet count: 9 × 10^9^/L	DXM (12 mg × 3 days) and rhTPO (15 000 IU × 14 days)	Platelet count increased to 30 × 10^9^/L
	December 2017	Thrombocytopenia re‐diagnosed	Platelet count: 12 × 10^9^/L	The patient was recommended splenectomy	The patient refused splenectomy
	January 2018	Low platelet count	Platelets decreased to 4 × 10^9^/L	HD‐DXM (40 mg × 4 days) Followed by IVIG (30 g × 5 days)	HD‐DXM improved the platelet count to 40 × 10^9^/L Platelet count did not rise any further with IVIG
	February 2018	Low platelet count	Platelets decreased to 8 × 10^9^/L	Splenectomy	No significant improvement in the platelet count
	12 March 2018	Thrombocytopenia	Platelet count: 5 × 10^9^/L	Amifostine (0.40 g day 1‐day 5 off 2 days, 4 cycles) and recombinant human interleukin‐11	Platelet count increased to 35 × 10^9^/L
	10 November 2018	Low platelet count	Platelet count: 3 × 10^9^/L	Platelet transfusion	Platelet count increased to 47 × 10^9^/L
	17 March, 2019	Low platelet count	Platelet count: 3 × 10^9^/L	Platelet transfusion	Platelet count increased to 66 × 10^9^/L
	5 May 2019	Patient was admitted to the hospital due to bleeding from the oral cavity and posterior pharyngeal wall	Platelet count: 4 × 10^9^/L	Platelet transfusion	Platelet count increased to 34 × 10^9^/L
	16 May 2019	Left temporal lobe and frontal occipital lobe haemorrhage occurred	Platelet count: 2 × 10^9^/L	HD DXM and IVIG	Platelet count increased to 114 × 10^9^/L Re‐examination showed better blood reports profile, and the patient was discharged
	April 2020	Patient admitted to the hospital due to oral and posterior pharyngeal haemorrhage	Platelet count: 3 × 10^9^/L	Oral Eltrombopag 75 mg q.d (from April 2020 to June 2021)	No significant improvement in the platelet count
	30 June 2021	Low platelet count	Platelet count: 3 × 10^9^/L	Avatrombopag 40 mg q.d	Platelet count increased to 48 × 10^9^/L
Case 4	8 December 2017	Thrombocytopenia	Low platelet count	IVIG treatment	Platelet count increased to normal but decreased again after discontinuation of the drug
	January 2018	Thrombocytopenia	Low platelet count	HD‐DXM, standard Pred, Danazol, Levamisole, and Interleukin‐11	The efficacy could not be maintained
	17 January 2018	Thrombocytopenia	Platelet count: 1 × 10^9^/L	RTX (100 mg, q.wk × 4 times)	No improvement in platelet counts
	February 2018	Thrombocytopenia	Platelet count: 1 × 10^9^/L	Splenectomy	No improvement in platelet counts
	2018–2022	Multiple hospital admissions for thrombocytopenia	Platelet count: 0–3 × 10^9^/L	Hormonal therapy and rhTPO	No satisfactory outcomes
	20 April 2022	Thrombocytopenia	Platelet count: 2 × 10^9^/L	SYK inhibitor (TQB3473 clinical trial)	On June 21, 2022, he withdrew from the group due to poor efficacy
	June–July 2022	Thrombocytopenia	Platelet count: 1–5 × 10^9^/L	Oral Chinese medicine	No significant improvement in platelet counts
	14 July 2022	Thrombocytopenia	Platelet count: 0 × 10^9^/L	Herombopag (7.5 mg, q.d)	Platelet count: 2 × 10^9^/L
	18 July 2022	Intracranial haemorrhage	Platelet count: 1 × 10^9^/L	HD‐DXM 40 mg × 4 days	Platelet count increased to 40 × 10^9^/L Follow‐up head CT scan showed thrombosis
	20 July 2022	Thrombocytopenia and thrombosis	Platelet count: 23 × 10^9^/L	Herombopag (7.5 mg, q.d)	Intracranial haemorrhage area expanded
	20 July 2022	Intracranial haemorrhage and patient went into coma	Platelet count: 23 × 10^9^/L	HD‐DXM (40 mg) and IVIG (1 g/kg); followed by surgery Post operative treatment: IVIG (0.4 g/kg/day × 5 days), HD‐DXM (40 mg × 4 days), rhTPO (15 000 IU × 14 days), and Herombopag (7.5 mg, q.d)	Platelet count: 178 × 10^9^/L but gradually reduced to 7 × 10^9^/L Post operative treatment did not increase the platelet count
	24 July–6 August 2022	Thrombocytopenia	Platelet count: 1 × 10^9^/L	IVIG 0.4 g/day × 5 days, HD‐DXM 40 mg × 4 days, rhTPO 15000 IU × 14 days, and Herombopag 7.5 mg q.d taken orally	No significant increase in platelets
	7 August 2022	Thrombocytopenia	Platelet count: 1 × 10^9^/L	Oral Avatrombopag (40 mg, q.d)	On 19 August 2022, platelet count increased to 143 × 10^9^/L but fluctuated around 100 × 10^9^/L after the dose adjustments Avatrombopag treatment was discontinued due to economic reasons resulting in a rapid decline in the platelet count to 1 × 10^9^/L No improvement was acheived after reinitiation of Avatrombopag

Abbreviations: DAC, decitabine; HD‐DXM, high dose dexamethasone; IU, international unit; IVIG, intravenous immune globulin; Pred, prednisolone; q.d, ever day; q.wk, every week; q12h, every 12 h; R‐CHOP, rituximab, cyclophosphamide, doxorubicin, vincristine sulfate and prednisone; R‐CHOP‐M rituximab, cyclophosphamide, doxorubicin, vincristine sulfate, prednisone and methotrexate; rhTPO, recombinant human thrombopoietin; RTX, rituximab; SYK, spleen tyrosine kinase; WBC, white blood cells.

**FIGURE 1:**
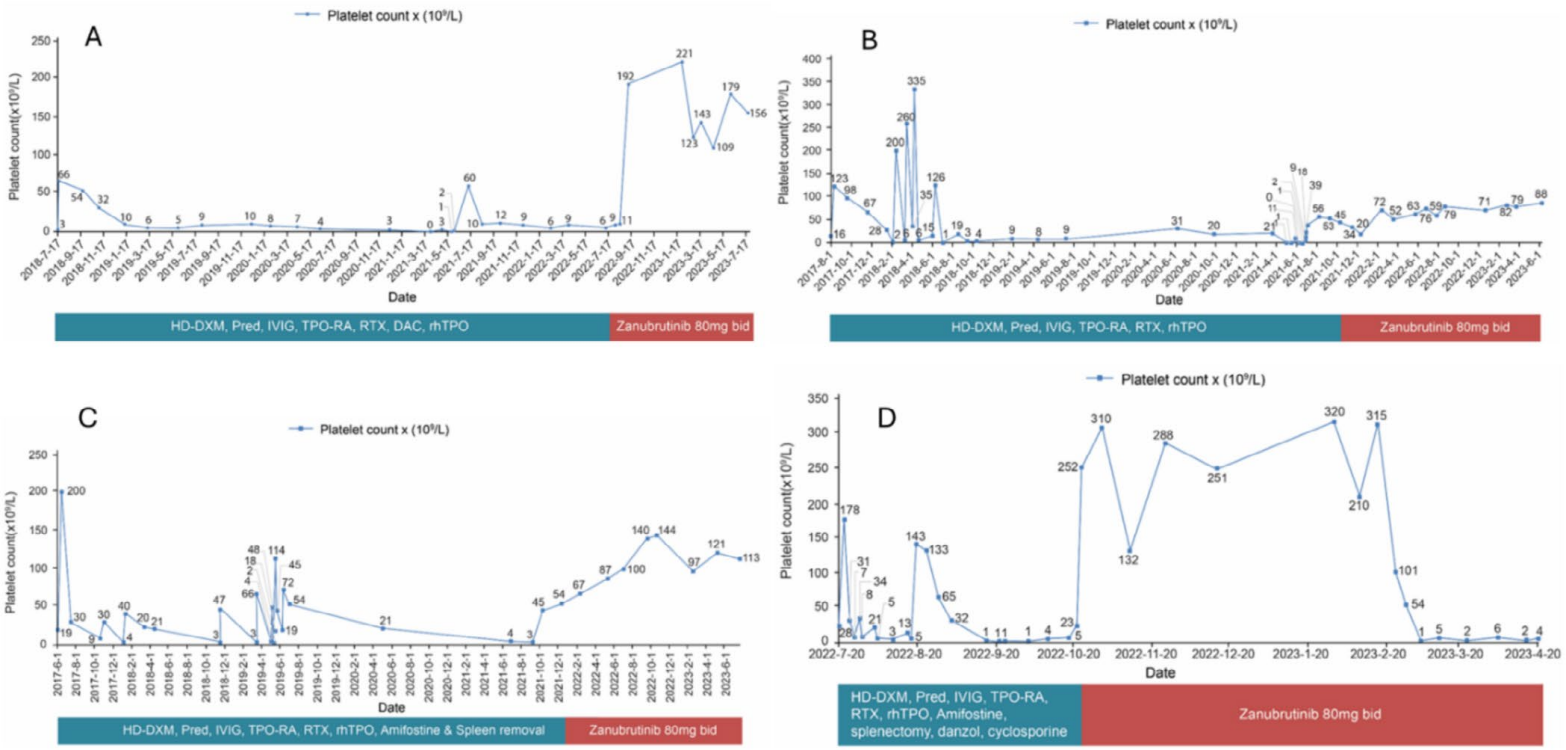
Improvements in platelet counts of ITP patients before and after Zanubrutinib treatment; (A) Patient 1, (B) Patient 2, (C) Patient 3 and (D) Patient 4.

The patient was initiated with Zanubrutinib (80 mg, bid) from August 2022. Patient outcomes from August 2022 to July 2023 after initiation of Zanubrutinib treatment has been described in Table 2. From February to July 2023, the number of days in which platelet values were maintained at 50 × 10^9^/L or higher was 334 days (Figure 1A).

**TABLE 2:** Zanubrutinib treatment outcome in the four included cases.

Case number	Status of patients before treatment with Zanubrutinib	Timeline	Outcome after treatment with Zanubrutinib
Case 1	Low platelet count: 11 × 10^9^/L White blood cells: 5.07 × 10^9^/L Haemoglobin: 62 g/L	15 August 2022	Gradual increase in platelet counts after initiation of Zanubrutinib (80 mg twice a day)
		7 September 2022	Platelet count increased to 192 × 10^9^/L
		26 February 2023	Platelet count: 123 × 10^9^/L. White blood cells count: 0.87 × 10^9^/L Neutrophil count: 0.1 × 10^9^/L Haemoglobin: 95 g/L Bone marrow smear showed markedly active myelodysplasia, visible megakaryocytes, and scattered platelets
		February–July 2023 20 July 2023	Fluctuation in platelet count (109 × 10^9^/L–221 × 10^9^/L) was reported Platelet values were maintained at 50 × 10^9^/L or higher for 334 days
Case 2	Low platelet count	26 June 2021	Zanubrutinib was initiated which increased platelet count to > 30 × 10^9^/L
		November 2021–June 2023	Fluctuation in platelet count (52 × 10^9^/L–88 × 10^9^/L) was reported As of 20 July 2023 the platelet values were maintained over 50 × 10^9^/L for 390 days
Case 3	Low platelet count	10 September 2021	Platelet count was 8 × 10^9^/L; Patient re‐admitted to hospital; Zanubrutinib (80 mg bid.) was initiated
		January 2022–April 2023	Platelet count fluctuated between 97 × 10^9^/L and 144 × 10^9^/L after the initiation of Zanubrutinib
		Till July 2023	Platelet count remained above > 50 × 10^9^/L continuously for 1045 days
Case 4	Platelet count decreased to 1 × 10^9^/L, after discontinuation of Avatrombopag	3 October 2022	Zanubrutinib was initiated, which improved platelet count to 252 × 10^9^/L and stabilised between 110 × 10^9^/L and 340 × 10^9^/L
		5 March 2023	Zanubrutinib was discontinued due to financial reasons; platelets dropped to 1 × 10^9^/L Patient died of intracranial haemorrhage on April 20, 2023 Platelet count had remained above > 50 × 10^9^/L for 120 days

Abbreviations: HD‐DXM, high dose dexamethasone; IVIG, intravenous immune globulin; Pred, prednisolone; RTX, rituximab.

### 2.2 Case 2

Patient 2, a 49‐year‐old male with thrombocytopenia for > 4 years, was admitted to the hospital on 1 July 2021. He presented with skin ecchymosis with a low platelet count of 16 × 10^9^/L in August 2017. Blood tests revealed anti‐glycoproteins (GPs) autoantibodies was positive, and the patient was consequently diagnosed with ITP with bone marrow examination. Treatment with HD‐DXM and IVIG increased the platelet count to normal levels.

The patient's medical history indicated a diagnosis of diffuse large B‐cell lymphoma, identified following a right epididymectomy due to right testicular swelling in January 2018. During follow‐up, the platelet count was 23 × 10^9^/L and PET‐CT revealed multiple metabolically active soft tissue nodules and masses at various sites, suggesting lymphoma infiltration. No abnormalities were detected in the bone marrow smear and immunophenotyping. In 7 February 2018, the patient underwent four courses of R‐CHOP‐M (rituximab, cyclophosphamide, doxorubicin, vincristine sulfate, prednisone and methotrexate) chemotherapy regimen. After two courses of chemotherapy, a CR was assessed through PET‐CT. From May 2018 to June 2021, the patient received various treatments to treat lymphoma and thrombocytopenia, the details of which are described in Table 1. In June 2021, due to limited effectiveness demonstrated by previous treatments, the treatment was modified to initiate Zanubrutinib (80 mg, bid, oral). Patient outcomes during the treatment with Zanubrutinib from June 2021 to June 2023 have been described in Table 2.

Throughout November 2021 to June 2023 period, the platelet count remained steadily above 50 × 10^9^/L for a continuous period of 390 days (Figure 1B). This case highlights the potential of zanubrutinib as a valuable therapeutic option for managing ITP, particularly in patients with a complex medical history that includes haematologic malignancies.

### 2.3 Case 3

Patient 3, a 31‐year‐old, dealing with thrombocytopenia for > 1.5 years received in‐patient care in March 2018. In June 2017, the patient's platelet count was 19 × 10^9^/L with scattered bleeding spots. Bone marrow puncture and other examinations were performed, and the patient was diagnosed with ITP. HD‐DXM (40 mg × 4 days) and rhTPO (15 000 IU × 7 days) raised platelets to 200 × 10^9^/L but were lowered to 30 × 10^9^/L in July 2017. Between July 2017 to June 2021, the patient received various treatments to manage ITP. The results of each treatment are described in Table 1. The patient's platelet count was 8 × 10^9^/L in September 2021, which led to his re‐hospitalisation. On 10 September 2021, Zanubrutinib (80 mg, bid) was initiated. The patient outcomes during the treatment with Zanubrutinib are described in Table 2. Till July 2023, the patient's platelet count remained above > 50 × 10^9^/L continuously for 1045 days (Figure 1C).

### 2.4 Case 4

Patient 4 was a 51‐year‐old female who was presented initially in March 2018 with a history of ITP for more than 11 years. The patient was diagnosed with ITP in 2011 and treated with Pred, HD‐DXM, Danazol, Caffeic acid tablets, Cyclosporine and so on but showed unsatisfactory outcomes. Bone marrow examinations showed active proliferation with megakaryocytes and normal chromosomes. Platelet count returned to normal after the commencement of IVIG but decreased after its discontinuation. Throughout 2017–2022, the patient received multiple treatment regimens to improve the platelet counts. The details of each treatment are described in Table 1. In October 2022, the patient initiated oral Zanubrutinib (80 mg, bid), as no improvement in platelet count was observed with the previous treatment. The patient outcome during treatment with Zanubrutinib is detailed in Table 2. The patient died in April 2023 due to an intracranial haemorrhage (Figure 1D).

## 3 Discussion and Literature Review

Over the decades, many scientific works have expanded the knowledge and understanding of autoimmune diseases including ITP. Owing to the ineffectiveness of current therapies against ITP and heterogeneity, the discovery of novel therapies is crucial to curbing this autoimmune disorder. Among various targets, BTK could be a viable target due to various functions, including regulation of B‐cell maturation, proliferation, differentiation and apoptosis. The therapeutic strategy of BTK inhibition has become crucial, especially in treating various haematologic malignancies including diffuse large B‐cell lymphoma, condylomatous lymphoma, and lymphoblastic leukaemia [23]. Furthermore, the kinase nature of BTK is essential in maintaining the homeostasis of many immune cells, which could effectively treat autoimmune disorders [24, 25, 26, 27, 28, 29]. However, few studies have reported the use of BTKis, including ibrutinib, rilzabrutinib, orelabrutinib, zanubrutinib and Zanubrutinib for ITP. In a study, Yu Tianshu et al. studied the effectiveness of BTKi in the inhibition of B‐cell proliferation, pro‐inflammatory cytokine secretion and plasma cell differentiation (activated via BCR pathway) and could inhibit FcTR‐mediated phagocytosis in ITP active mice model [30]. This preclinical study provides a strong basis for the efficacy of BTKi against ITP disease. In a clinical setup, Hampel et al. analysed patients with chronic lymphocytic leukaemia (CLL) and treated them with ibrutinib. Among the 193 patients with CLL, 29 CLL patients had autoimmune cytopenias (AICs) prior to ibrutinib treatment. Patients who received ibrutinib did not show any major variation in EFS or OS based on their prior history of AIC. This indicates that ibrutinib possibly nullified the unfavourable outcome associated with AIC [31].

Rilzabrutinib, an oral, reversible, covalent BTKi used for treating immune‐mediated disorders, is currently being studied in a phase 3 trial and was evaluated for its inhibitor effect in 60 patients with relapsed/refractory ITP. The median platelet count was reported to be 15 × 10^9^/L with a duration of 6.3 years and received 4 lines of ITP therapy. On a median of 167.5 days of treatment, 24 patients (40%) treated with rilzabrutinib (400 mg, bid) achieved a platelet response of 50 × 10^9^/L at 11.5 days. This study indicates the safety and clinical feasibility of BTKi for the treatment of ITP [10].

Orelabrutinib is another BTKi used for inhibiting multiple signaling pathways in immune‐mediated disorders. In a preclinical evaluation, orelabrutinib exhibited a significant reduction/inhibition of CD69 & CD86 (expressed in isolated polymorphonuclear cells of both patients with ITP and healthy humans). The administration of orelabrutinib significantly increased the platelet counts in the ITP mice model [30]. In another randomised open‐label phase II study, orelabrutinib was evaluated for its safety, efficacy and tolerability in persistent/chronic ITP patients. The proportion of patients who achieved at least two consecutive platelet counts ≥ 50 × 10^9^/L without rescue medication in the 4 weeks before the elevated platelet count was considered as the primary endpoint. Overall, patients who achieved the primary endpoint were 36.4% (12/33) of which 40% (6/15) were from the 50 mg arm, and 22.2% (4/18) were from the 30 mg arm. Two patients achieved the endpoint after transferring to the 50 mg arm. This study showed that the patients treated with orelabrutinib exhibited better efficacy especially those who were previously treated with GC/IVIGs [32].

Zanubrutinib is a new‐generation BTKi that acts by covalently binding to the cysteine at site 481 of the BTK protein and thereby inhibiting its tyrosine phosphorylation at site 223. It is currently approved for the treatment of adult mantle cell lymphoma (MCL), adult chronic lymphocytic leukaemia (CLL)/small lymphocytic lymphoma (SLL) and Wahl's macroglobulinemia (WM). In our previous case study, we reported a case of a 15‐year‐old patient with Evans syndrome with platelet elevation after treatment with zanubrutinib, which could be used for the treatment of ITP [33]. In China, clinical studies on zanubrutinib and eltrombopag were considered as second‐line treatments for refractory ITP (NCT05369377). Many clinical trials evaluating zanubrutinib as montherapy (NCT05214391)/(NCT05279872)/(NCT05199909) or in combination with high‐dose dexamethasone (NCT05369364) or rituximab (ChiCTR2200057058) were ongoing with more focus on its effectiveness towards ITP. It could provide new therapeutic horizons for patients with relapsed/refractory ITP.

In the present case study, all four patients who were treated with different ITP treatment regimens reported significant improvement in platelet counts following treatment with Zanubrutinib. These patients did not respond to splenectomy or other interventions. Overall, one patient achieved CR, and two patients achieved partial response (PR) with sustained remission after Zanubrutinib intervention. The blood analysis revealed that the platelet counts of three patients were maintained above 50 × 10^9^/L for 1045, 390 and 334 days, respectively. One patient died of intracranial haemorrhage due to platelet decline after discontinuation of the drug. However, the deceased patient stayed in a sustained remission for 120 days before treatment discontinuation. During the treatment, no significant abnormalities in liver or kidney functions were reported, suggesting that Zanubrutinib showed a favourable safety profile in these cases. These findings suggest that BTK inhibitors, such as Zanubrutinib, may offer a new therapeutic option for patients who have exhausted traditional treatment options. However, several gaps remain in our understanding of the long‐term efficacy and safety of Zanubrutinib for ITP. The study involves only four patients, limiting the generalisability of the findings. A larger cohort would yield more reliable data on the effectiveness and safety of the treatment. The previous treatment regimen variations may further complicate the analysis of Zanubrutinib's effectiveness as a monotherapy. In addition, although no significant abnormalities in liver and kidney function were reported during treatment, long‐term monitoring for adverse effects of BTK inhibitors remains crucial to understand potential risks associated with prolonged use.

## 4 Conclusion

Zanubrutinib demonstrated sustained remission and maintained platelet levels. These findings indicate that Zanubrutinib can be a promising option for treating ITP. However, treatment with BTK inhibitors is still in the exploratory phase and requires further investigation through clinical trials to accurately assess its efficacy and safety in chronic or persistent ITP.

## Author Contributions

Hu Zhou designed the study. Bingjie Ding analysed the data and wrote the manuscript. Mengjuan Li, Xuewen Song, Yuanyuan Zhang, Ao Xia and Jingyuan Liu collected and collated the clinical data. Hu Zhou and Liu Liu reviewed the manuscript. All authors have read and agreed to the published version of the manuscript.

## Ethics Statement

The study was approved by the Medical Ethics Committee of Henan Cancer Hospital.

## Consent

Informed consent was obtained from all patients enrolled in the study.

## Conflicts of Interest

The authors declare no conflicts of interest.

## Data Availability

The authors have nothing to report.
